# Suspected Acute Pancreatitis With Peripancreatic Fluid Collection in an Adolescent: A Possible Association With Energy Drink Overconsumption

**DOI:** 10.1002/ccr3.72458

**Published:** 2026-04-06

**Authors:** Hafiza Tooba Siddiqui, Muhammad Umar, Mirza Mohammad Ali Baig, Hasnain Wajeeh Saqib, Mohammed Hammad Jaber Amin

**Affiliations:** ^1^ Jinnah Sindh Medical University Karachi Pakistan; ^2^ Khairpur Medical College Khairpur Mir's Pakistan; ^3^ Islamic International Medical College, Riphah International University Rawalpindi Pakistan; ^4^ Faculty of Medicine Alzaiem Alazhari University Khartoum Sudan

**Keywords:** adolescent case report, energy drink‐induced pancreatitis, pancreatic injury, peripancreatic fluid collection

## Abstract

Acute pancreatitis is uncommon in adolescents without underlying risk factors. Excessive consumption of energy drink–type beverages has been rarely reported as a possible contributing factor, although causality remains uncertain. We report a 14‐year‐old male who presented with severe epigastric pain radiating to the back and recurrent vomiting. He had a history of consuming over three bottles of a carbonated energy–type drink daily for eight months. Laboratory evaluation revealed elevated amylase (202 U/L) and lipase (245 U/L), and imaging identifies a peripancreatic fluid collection. Common causes of pancreatitis including gallstones, hypertriglyceridemia, and hypercalcemia were assessed. The patient received conservative management including bowel rest, intravenous fluids, analgesics, antiemetics, and antibiotics, with gradual clinical improvement. A possible association between chronic excessive energy drink–type beverage consumption and pancreatic injury is described in this adolescent case. Awareness, education, and preventive measures are essential to reduce the incidence of such cases. Evaluation included assessment for common causes of pancreatitis including gallstones, hypertriglyceridemia, hypercalcemia, medication exposure, abdominal trauma, and metabolic disorders, although complete exclusion of rare etiologies was limited by resource constraints.

## Key Clinical Message

Excessive chronic consumption of energy drink–type beverages may represent a potential contributory factor in pancreatic injury, including suspected acute pancreatitis and peripancreatic fluid collections, in adolescents without traditional risk factors. Clinicians should inquire about energy drink intake when evaluating unexplained pancreatic pathology in pediatric and adolescent patients.

## 1 Introduction

Acute pancreatitis is an inflammatory condition of the pancreas characterized by abdominal pain and elevation of pancreatic enzymes. Although less common in children and adolescents than in adults, its incidence in pediatric populations has increased in recent years. The most frequent causes include gallstones, medications, infections, metabolic disorders, trauma, structural abnormalities, and genetic predisposition. Despite thorough evaluation, a proportion of cases remain idiopathic [1].

Peripancreatic fluid collections are among the early local complications of acute pancreatitis. These collections typically develop within the first four weeks of inflammation and lack a well‐defined fibrous wall.^2^ In contrast, pancreatic pseudocysts represent later sequelae that arise after maturation of the collection wall, usually several weeks following the initial episode. Imaging modalities such as ultrasonography and contrast‐enhanced computed tomography play a central role in identifying these collections and distinguishing them from other cystic lesions of the pancreas [2].

Energy drink consumption has risen markedly worldwide, particularly among adolescents and young adults [3]. These beverages often contain high concentrations of caffeine, sugars, and stimulatory additives, such as taurine and guarana. While cardiovascular and neurological adverse effects are well documented, reports linking excessive energy drink intake to pancreatic injury remain rare and are largely limited to isolated case reports. Several adult cases have described acute pancreatitis occurring after heavy consumption of energy drinks, with symptom resolution following discontinuation of the beverages, suggesting a possible association [4]. However, reports in pediatric or adolescent populations remain extremely limited.

We report a case of suspected acute pancreatitis with an associated peripancreatic fluid collection in a previously healthy adolescent with chronic excessive consumption of energy drink–type beverages. This case highlights a possible association between heavy energy drink intake and pancreatic inflammation in younger populations.

## 2 History

A 14‐year‐old male patient presented to the emergency department with a 2‐day history of epigastric pain associated with vomiting. His weight was 45 kg. The epigastric pain was described as sharp and severe, which began suddenly three days before presentation. The pain radiated to the back and was partially relieved by intravenous analgesics. It was aggravated by meals and associated with more than two episodes of non‐bilious, projectile vomiting. The patient had a long‐standing history of heavy intake of soft drinks, having been consuming over three bottles of energy drink–type (Sting) per day for eight months. Each 250 mL bottle contains approximately 34 mg caffeine and 34 g sugar. Three bottles per day yield approximately 102 mg caffeine (~2.3 mg/kg/day) and 102 g sugar daily. The recommended safe upper intake of caffeine for adolescents is ~2.5 mg/kg/day, suggesting this patient's intake chronically approached or potentially exceeded safe limits. No objective biomarkers of caffeine exposure were assessed due to resource limitations. He had no history of previous medical or surgical illness, without any prior gastrointestinal illness. No history of pancreatic disease, diabetes, or other such conditions was elicited in the family. There was no history of abdominal trauma, medication use, or known genetic conditions predisposing to pancreatitis. Autoimmune markers and magnetic resonance cholangiopancreatography (MRCP) for ductal anomalies were not performed also due to the resource limited setting.

On physical examination, the patient was afebrile, with the vital signs indicating a blood pressure of 100/60 mmHg, pulse of 71 beats/min, respiratory rate of 16 breaths/min, SpO2 of 99%, and random blood sugar (RBS) of 60 mg/dL. The hypoglycemia was corrected with intravenous dextrose‐containing fluids, and serial glucose monitoring was performed, after which glucose levels normalized. On abdominal examination, there was mild epigastric tenderness, with ill‐defined fullness and ill‐defined margins palpable in the region, indicating probable pancreatic involvement. No evidence of peritoneal irritation was observed.

## 3 Differential Diagnosis, Investigations, and Treatment

Based on the clinical presentation, the differential diagnosis included acute pancreatitis with peripancreatic fluid collection formation, gastritis, peptic ulcer disease, and biliary pathology. Initial laboratory investigations included a complete blood count (CBC), C‐reactive protein (CRP), amylase, lipase, and aspartate aminotransferase (AST). Results showed hemoglobin 13.7 g/dL, total leukocyte count 9.7 × 10^9^/L, CRP 1.3 mg/L, amylase 202 U/L, lipase 245 U/L, and AST 60 U/L. With the upper limit of normal (ULN) for lipase being ~60 U/L, the measured value of 245 U/L would exceed 3 times the ULN. Per standard pediatric criteria, diagnosis requires ≥ 2 of: (1) characteristic abdominal pain, (2) lipase/amylase ≥ 3× ULN, (3) imaging consistent with pancreatitis. With CT showing no active pancreatitis, this patient met criterion 1 and criterion 2. Therefore, the diagnosis was framed as suspected acute pancreatitis based on clinical and biochemical basis. Detailed laboratory results are presented in Table 1.

**TABLE 1:** Initial laboratory investigations.

Investigation	Value	Reference range
CBC		
Hb	13.7 g/L	3.5–17.5 g/dL (men) 12–16 g/dL (women)
TLC	9.7 × 10^9^/L	4–10 × 10^9^/L
PLT	371 × 10^9^/L	150–400 × 10^9^/L
CRP	1.3 mg/L	< 3 mg/L
Amylase	**202 U/L**	30–110 U/L
Lipase	**245 U/L**	13–60 U/L
AST	**60 U/L**	0–35 U/L
ALT	18 U/L	0–45 U/L
ALP	**212 IU/L**	44–147 IU/L
Serum sodium	140 mEq/L	135–145 mEq/L
Serum potassium	3.8 mEq/L	3.5–5.0 mEq/L
Serum chloride	101 mEq/L	96–106 mEq/L
LDH	250 U/L	140–280 U/L
Urea	**27 mg/dL**	7–20 mg/dL
Creatinine	**0.59 mg/dL**	0.7–1.3 mg/dL (men) 0.6–1.1 mg/dL (women)
PT	11.0 s	11–13.5 s
INR	1.08	0.8–1.1
Total cholesterol	172 mg/dL	< 200 mg/dL
LDL cholesterol	**106 mg/dL**	< 100 mg/dL
HDL cholesterol	**40.8 mg/dL**	> 60 mg/dL
Triglycerides	116 mg/dL	< 150 mg/dL
FBS	71 mg/dL	< 100 mg/dL

*Note:* Abnormal values are in bold characters.

Abbreviations: ALP, alkaline phosphatase; ALT, alanine aminotransferase; AST, aspartate aminotransferase; CBC, complete blood count; CRP, c‐reactive protein; FBS, fasting blood sugar; Hb, hemoglobin; INR, international normalized ratio; LDH, lactate dehydrogenase; PLT, platelet count; PT, prothrombin time; TLC, total leukocyte count.

**FIGURE 1:**
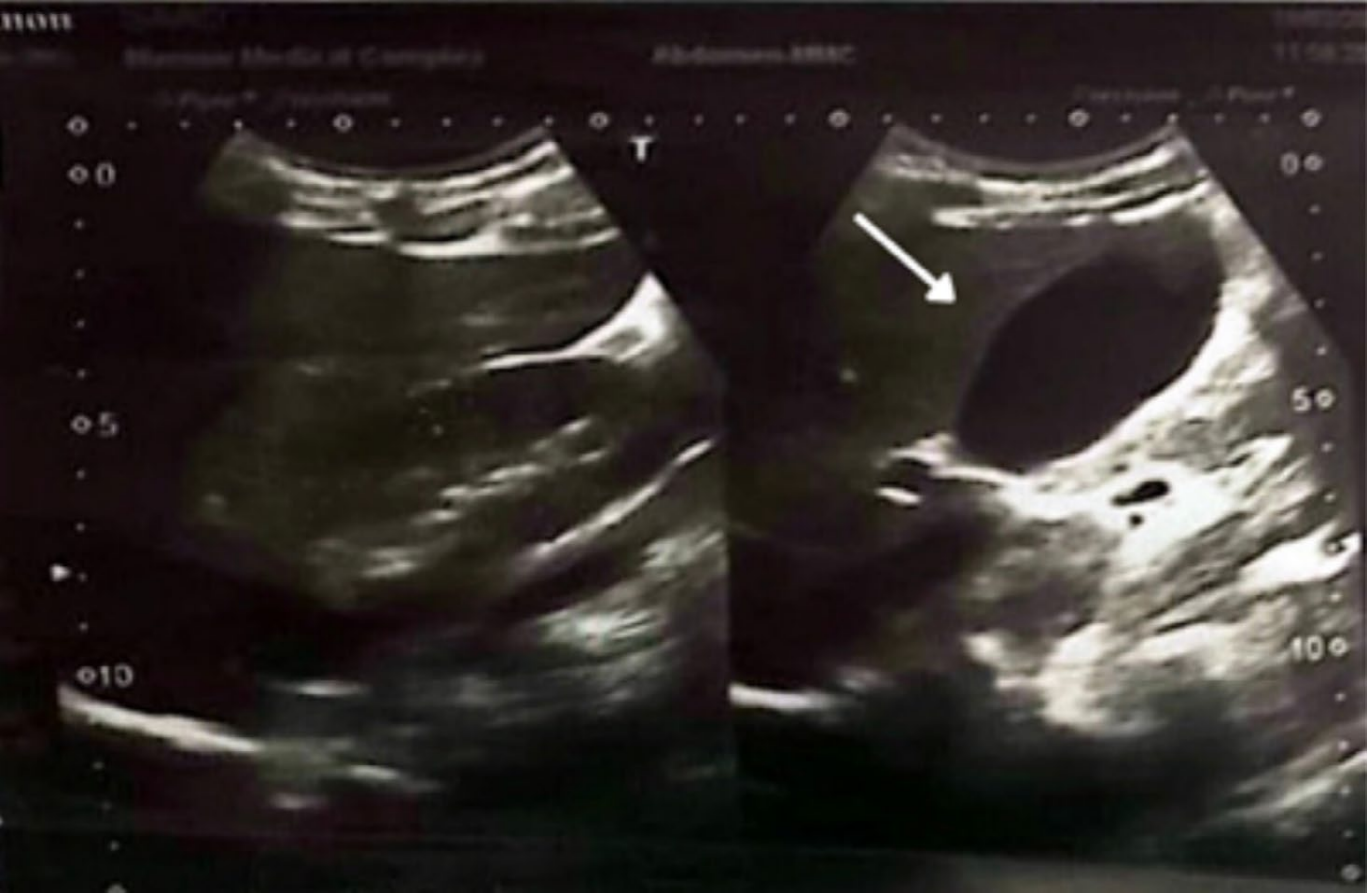
Ultrasound for the whole abdomen: A cystic area measuring 4.0 × 1.3 cm in the pancreas arising from the head towards the body, suggestive of peripancreatic fluid collection.

Abdominal ultrasonography revealed a thick‐walled linear peripancreatic fluid collection measuring approximately 4.0 × 1.3 cm within the pancreatic head and neck, consistent with a peripancreatic fluid collection (likely acute peripancreatic fluid collection given the early presentation) (Figure 1). The remainder of the abdominal scan was unremarkable, with no evidence of biliary tract disease or free fluid.

The patient was initially kept nil per os (NPO) for symptomatic control; however, current pediatric pancreatitis guidelines generally favor early oral or enteral feeding as tolerated, and diet was gradually reintroduced once symptoms improved. Intravenous fluids were administered for rehydration, and analgesics were provided for pain control. Given the low presenting RBS, blood glucose levels were monitored frequently (RBS three times daily).

Intravenous ceftriaxone (1 g twice daily) was initiated due to institutional clinical practice and concern for possible secondary infection, although routine prophylactic antibiotics are generally not recommended for sterile peripancreatic fluid collections. Proton pump inhibitor therapy (Omeprazole IV 20 mg daily) was started to suppress gastric acid secretion. Antiemetics (metoclopramide intravenously at 0.1–0.2 mg/kg per dose) and analgesics (Paracetamol intravenously at a dose of 15 mg/kg per dose every 6–8 h) were administered. Supportive intravenous fluids, including Ringer's lactate and normal saline, were continued.

**FIGURE 2:**
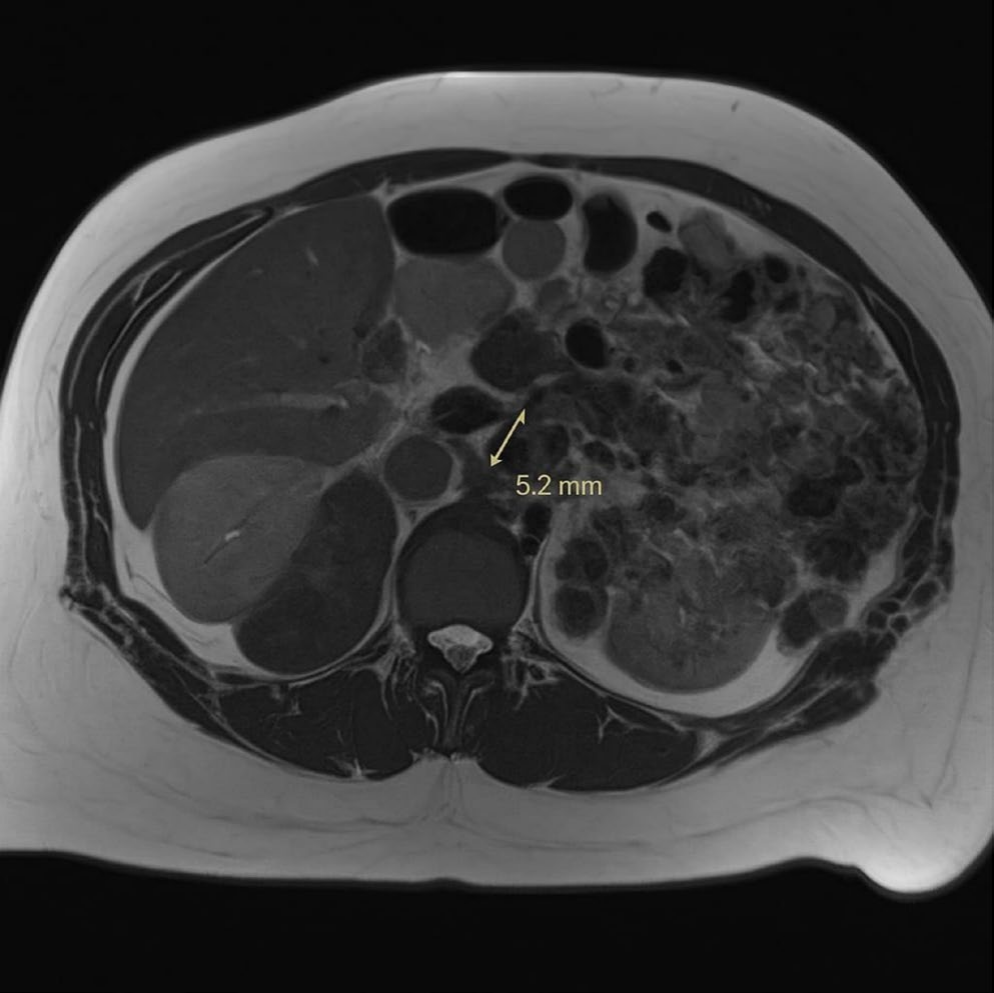
Contrast‐enhanced axial CT scan of the abdomen: A rounded, well‐defined, low attenuation fluid collection in the left upper quadrant, consistent with peripancreatic fluid collection.

**FIGURE 3:**
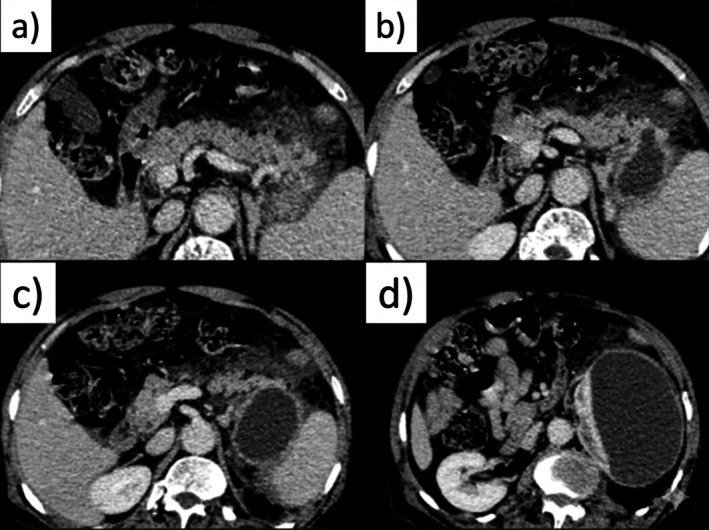
Contrast‐enhanced CT scan of the abdomen in axial view demonstrating a peripancreatic fluid collection (a–d). (a–d) Serial axial CT images show a well‐defined, low‐attenuation fluid collection in the lesser sac, located posterior to the stomach and anterior to the left kidney. The lesion is consistent with a peripancreatic fluid collection, with mild mass effect on surrounding structures and without evidence of pancreatic necrosis.

A contrast‐enhanced computed tomography (CT) scan of the abdomen reaffirmed the presence of a small peripancreatic fluid collection without evidence of pancreatic necrosis or mature cyst wall formation, consistent with an early inflammatory fluid collection (Figures 2 and 3). No other abdominal pathology was identified.

## 4 Conclusion and Results (Outcome and Follow‐Up)

The patient demonstrated steady clinical improvement with conservative management and was discharged after 14 days in stable condition. The patient reported complete resolution of abdominal pain at discharge and was able to tolerate oral feeding without recurrence of vomiting. He was counseled extensively regarding cessation of energy drink consumption and advised close outpatient follow‐up. Unfortunately, the patient was lost to follow‐up after hospital discharge despite advice for outpatient reassessment and repeat imaging. No formal outpatient review was possible and no repeat clinical, laboratory, or imaging assessment could have been obtained. Consequently, it is not possible to confirm the resolution or maturation of the peripancreatic fluid collection into a true pseudocyst or verify the normalization of pancreatic enzyme levels. This represents a significant limitation of this report. This case, however, does underscore a favorable outcome with early recognition, supportive management, and elimination of the suspected contributory factor.

## 5 Discussion

We present an uncommon case of a 14‐year‐old male with a 2‐day history of severe epigastric pain radiating to the back, accompanied by recurrent vomiting. The pain intensified following meals and was partially alleviated by analgesics. The patient disclosed excessive soft drink consumption, reporting an intake of over three bottles of Sting daily for the preceding eight months, with no notable personal or family medical history. On clinical examination, he was hemodynamically stable, afebrile, and exhibited mild epigastric tenderness without evidence of peritoneal irritation. Imaging and clinical assessment identified a peripancreatic fluid collection consistent with early inflammatory fluid accumulation rather than a mature pseudocyst. Conservative management was initiated with bowel rest, intravenous fluids, analgesics, antiemetics, and antibiotics, leading to discharge in stable condition after 14 days.

Recurrence of acute pancreatitis is influenced by the precipitating cause. These observations emphasize the critical role of early recognition and treatment of the underlying factor to minimize subsequent episodes [5].

Energy drinks are extensively promoted as beverages that enhance both physical endurance and cognitive capacity [6]. According to the U.S. Food and Drug Administration (FDA), they are defined as liquid formulations typically containing caffeine, either alone or in combination with other substances. Common constituents include high concentrations of caffeine and sugars, as well as legally permitted stimulants such as guarana, taurine, and L‐carnitine. These ingredients contribute to increased alertness, focus, and energy levels but may also raise blood pressure, heart rate, and respiratory rate. Consequently, energy drinks are marketed as agents that heighten mental acuity and physical performance [7]. Evidence indicates that consumption patterns of energy drinks are highly variable yet widespread, particularly among younger demographics [8]. A systematic review estimated the global prevalence of energy drink consumption to be 54.7% [9]. Furthermore, data from the European Food Safety Authority (EFSA) revealed that intake is most common among adolescents (68%), followed by adults (30%) and children (18%). Among underage consumers, high chronic consumption rates were 12% in adolescents and 16% in children, while high acute consumption was reported at 12% [10].

Energy drinks, primarily due to their caffeine content—often combined with glucose or other active ingredients—have been reported to enhance endurance, alertness, memory, concentration, mood, and reaction time in various contexts, particularly during fatigue or sleep deprivation. Documented benefits include prolonged time to exhaustion, greater mental focus, and reduced perception of fatigue [6]. Conversely, some investigations have demonstrated no significant influence on anaerobic power, muscular strength, oxygen uptake, or perceived exertion [6]. Despite these potential advantages, energy drink intake is linked to several adverse health outcomes. Cardiovascular complications include tachycardia, hypertension, arrhythmias, myocardial infarction, aneurysm formation, and endothelial dysfunction. Neurological and psychiatric consequences involve caffeine intoxication (manifesting as anxiety, insomnia, or headaches), psychiatric disturbances, aggressive behavior, seizures, stroke, and hallucinations; moreover, caffeine–taurine–guarana interactions may promote neuronal apoptosis. Gastrointestinal and metabolic derangements arise from excessive sugar and caffeine intake, contributing to obesity, diabetes mellitus, altered gut microbiota, insulin resistance, hepatic injury, and renal impairment. Dental complications include enamel erosion, hypersensitivity, and caries development [6].

Multiple case reports have documented an association between excessive energy drink intake and the onset of pancreatitis or other gastrointestinal manifestations. One report described a 46‐year‐old male with several comorbidities—including type 2 diabetes, hypertension, hyperlipidemia, hyperuricemia, nonalcoholic fatty liver disease (NAFLD), and a history of bilateral nephrectomies—who developed progressive epigastric pain, nausea, and vomiting after consuming two to three cans of *Monster Energy* daily over a four‐month period. Imaging revealed diffuse hepatic steatosis. Upon discontinuation of energy drinks, his symptoms completely resolved within a month despite the continuation of his regular medications, implicating energy drinks as the precipitating factor [11]. Another case involved a 62‐year‐old man with chronic pancreatitis, atrial fibrillation, cardiomyopathy, chronic kidney disease (CKD), and a history of gastric bypass surgery, who presented with severe abdominal pain, nausea, and vomiting following heavy energy drink use. Imaging confirmed chronic pancreatitis without acute changes, and his symptoms subsided after supportive care, suggesting that energy drink consumption exacerbated his underlying condition [12]. Similarly, a 29‐year‐old previously healthy male developed acute pancreatitis after ingesting five to six energy drinks per day, including seven 16‐oz cans the day prior to admission. Laboratory evaluation revealed marked lipase elevation, and imaging confirmed pancreatitis without evidence of other etiologies. Symptomatic improvement following cessation of energy drink use strongly indicated a causal relationship [13]. Another case featured a 19‐year‐old man harboring a *SPINK1* gene mutation who experienced recurrent episodes of acute pancreatitis—ten hospitalizations in one year—associated with regular consumption of *Red Bull* and *Full Throttle* energy drinks. No further attacks occurred within seven months after cessation, fulfilling the criteria for drug‐induced pancreatitis and suggesting energy drinks as a precipitating factor in genetically susceptible individuals [4]. Finally, a 27‐year‐old post‐cholecystectomy male developed recurrent acute pancreatitis after consuming up to six energy drinks daily, with no alternative etiologies identified, making energy drinks the probable cause [14].

Our case is a 14‐year‐old male who developed a peripancreatic fluid collection subsequent to excessive soft drink consumption, suggesting a possible temporal association between excessive energy drink–type beverage intake and pancreatic injury, although causality cannot be established. The patient presented with acute epigastric pain radiating to the back and episodes of vomiting, consistent with classical manifestations of pancreatic inflammation, in line with previously documented cases [11, 12, 13]. Unlike most reported adult cases, this patient was adolescent, previously healthy, and had no underlying comorbidities such as diabetes, hypertension, NAFLD, atrial fibrillation, or non‐ischemic cardiomyopathy, which were noted in other studies [12, 13]. Moreover, in contrast to a reported 19‐year‐old case with a SPINK1 mutation [4], no genetic predisposition was identified in our patient. The temporal association between prolonged soft drink intake and symptom onset, coupled with symptom resolution following cessation, parallels findings in adult cases where discontinuation of energy drinks resulted in clinical improvement [11, 12, 13].

Several mechanisms have been proposed to explain how excessive energy drink consumption could contribute to pancreatic injury. High caffeine intake may stimulate pancreatic enzyme secretion and increase metabolic activity within pancreatic acinar cells, potentially predisposing to inflammatory injury. Taurine and other additives present in many energy drinks may influence intracellular calcium signaling, a key pathway involved in premature activation of pancreatic digestive enzymes. In addition, the high sugar content of these beverages may contribute to metabolic stress and transient hypertriglyceridemia, both of which are recognized as risk factors for pancreatic inflammation. Although these mechanisms provide biological plausibility, current evidence remains limited and largely derived from experimental data and isolated clinical observations.

Alternative explanations for pancreatic or peripancreatic cystic lesions in pediatric patients should also be considered, including congenital pancreatic cysts, duodenal duplication cysts, choledochal cysts, pancreatic duct anomalies, and rarely cystic pancreatic neoplasms. Although imaging findings and clinical context in this case favored an inflammatory fluid collection, complete exclusion of these rare entities was limited by the absence of advanced imaging such as MRCP.

Energy drink (ED) consumption poses notable risks for individuals with preexisting health conditions [14]. Elevated cardiovascular disease (CVD) risk in men with nonalcoholic fatty liver disease (NAFLD) has been linked to greater intake of calorie‐rich energy drinks and alcohol, coupled with increased insulin resistance. Caffeine acutely elevates blood pressure, imposing additional cardiovascular burden and raising the risk of arrhythmias, particularly in older adults and hypertensive patients. Evidence indicates that energy drinks can induce hypertension when compared with placebo [15]. Caffeine acts through vasoconstriction, stimulation of skeletal and cardiac muscles, alteration of insulin sensitivity, and modulation of gene expression. High doses can increase urinary and sweat excretion, potentially affecting electrolyte homeostasis. Consumption up to 500 mg/day is generally considered safe, whereas doses of 4–12 mg/kg may cause anxiety, jitteriness, headache, or fatigue. Severe intoxication may lead to tachycardia, tremor, seizures, arrhythmias, cerebral edema, or death, with additional complications including vomiting, abdominal pain, hypokalemia, hallucinations, elevated intracranial pressure, stroke, paralysis, rhabdomyolysis, altered consciousness, rigidity, and arrhythmias [16]. For hypertensive individuals, the pressor effects of energy drinks can represent serious health threats. Moreover, these beverages contain high levels of sugars—primarily sucrose, glucose, or high‐fructose corn syrup—which can increase obesity and type 2 diabetes risk. High sugar intake may adversely alter gut microbiota, reducing activity, diversity, and gene expression, thereby contributing to metabolic syndrome. Acute caffeine intake also decreases insulin sensitivity, potentially explaining transient hyperglycemia following consumption [6]. In patients with diabetes, energy drinks may worsen insulin resistance and glycemic control, highlighting the amplified risk in susceptible populations. Overall, energy drink consumption may exacerbate cardiovascular, metabolic, and glycemic dysfunction, especially among individuals with conditions such as hypertension, NAFLD, or diabetes.

Genetic factors contribute to the variability observed in individual caffeine consumption and in its physiological effects. Variations in pharmacodynamic and pharmacokinetic genes have been linked to differential caffeine responses [17]. Additionally, genetics influence the consumption of sugar‐containing beverages, with some individuals exhibiting greater difficulty resisting high‐sugar drinks, potentially due to heightened taste perception or enhanced reward experiences [7]. Evidence also suggests a genetic influence on caffeine's effects, as variations in the CYP1A2 and ADORA2A genes may alter the relationship between caffeine intake and brain‐related outcomes [18].

Energy drinks comprise a variety of active ingredients in addition to caffeine, which may produce differential effects among users. Taurine, abundant in cardiac and skeletal muscle, contributes to neuromodulation, membrane stability, and intracellular calcium regulation. It exerts anti‐arrhythmic effects by influencing K^+^ and Na^+^ currents and may support neuroprotection and neurotransmission. When ingested with caffeine, taurine may improve focus and reaction speed, though supporting studies are limited. Carnitine, including L‐carnitine and acetyl‐L‐carnitine, is vital for energy metabolism as it mediates the transport of long‐chain fatty acids into mitochondria for ATP production and is obtained from both dietary sources and endogenous synthesis. Guarana, a naturally caffeine‐rich plant, contains additional stimulants, such as theobromine and theophylline, enhancing the overall stimulatory impact of energy drinks. Its caffeine content is often unreported due to herbal supplement regulations. Collectively, these components contribute to the cardiovascular, cognitive, and stimulant effects of energy drinks like Red Bull, though clinical evidence on their combined cognitive efficacy remains limited [9].

## 6 Limitations

This case report has several important limitations. First, as a single case, it cannot establish causality. Temporal association between energy drink consumption and pancreatitis does not equate to causal inference. Second, IgG4 and MRCP for ductal anomalies were not performed due to resource limitation. Lack of these investigations cannot let us exclude rare causes such as congenital ductal anomalies or cystic pancreatic lesions. Third, caffeine exposure was based solely on self‐reported consumption without objective biomarker confirmation. Fourth, the missing imaging criteria on CT scan do not allow us to definitely confirm the diagnosis of acute pancreatitis. Finally, no outpatient follow‐up could have been documented. This loss to follow‐up fundamentally limits the ability to validate both the final diagnosis and the reported clinical outcome and is a critical limitation of this case report.

## 7 Conclusion

This case highlights a possible association between the overconsumption of energy drink–type beverages and pancreatic injury. Healthcare providers should consider energy drink overconsumption as a potential contributing exposure in patients presenting with acute epigastric pain and vomiting, especially when common causes such as gallstones, alcohol use, or genetic factors are absent. Public health initiatives should aim to limit access and raise awareness among children and adolescents about the gastrointestinal and metabolic risks linked to chronic high intake. Targeted educational interventions in schools, households, and youth‐focused media may promote safer alternatives. Furthermore, enhanced labeling regulations on caffeine and sugar content, accompanied by consumption warnings, could mitigate such risks and safeguard vulnerable populations.

## Author Contributions

**Hafiza Tooba Siddiqui:** conceptualization, data curation, investigation, methodology, project administration, supervision, validation, writing – original draft, writing – review and editing. **Muhammad Umar:** conceptualization, data curation, validation, visualization, writing – original draft, writing – review and editing. **Mirza Mohammad Ali Baig:** supervision, writing – original draft, writing – review and editing. **Hasnain Wajeeh Saqib:** writing – review and editing. **Mohammed Hammad Jaber Amin:** writing – review and editing.

## Funding

The authors have nothing to report.

## Ethics Statement

This case report was conducted in accordance with the ethical standards of the institutional review board. Formal approval was waived for this retrospective case study as per institutional guidelines.

## Consent

Written informed consent was obtained from the patient and legal guardian for the publication of this case report, including de‐identified clinical details and images.

## Conflicts of Interest

The authors declare no conflicts of interest.

## Data Availability

Data sharing not applicable to this article as no datasets were generated or analysed during the current study.
